# Treatment-Free Survival and the Pattern of Follow-Up Treatments After Curative Prostate Cancer Treatment, a Real-World Analysis of Big Data from Electronic Health Records from a Tertiary Center

**DOI:** 10.3390/jpm16010022

**Published:** 2026-01-04

**Authors:** Fréderique B. Denijs, Sebastiaan Remmers, Leonard P. Bokhorst, Roderick C. N. van den Bergh, Monique J. Roobol

**Affiliations:** 1 Department of Urology, Erasmus MC Cancer Institute, University Medical Center Rotterdam, 3015 Rotterdam, The Netherlands; 2 Department of Radiotherapy, Haaglanden Medical Center, 2512 The Hague, The Netherlands

**Keywords:** prostate cancer, real-world data, electronic health records, treatment-free survival, curative therapy, radical prostatectomy, brachytherapy, radiotherapy, treatment sequences, medication profiles

## Abstract

**Background:** Prospective trials provide robust evidence for prostate cancer (PCa) treatment but often include highly selective populations, limiting generalizability. Real-world data (RWD) can address these gaps and inform personalized care. **Objectives**: This study aimed to evaluate treatment-free survival (TFS) and secondary treatment sequences after initial curative therapy for PCa using electronic health record (EHR) data and to analyze associated medication profiles. **Methods**: We studied 3024 patients treated with radical prostatectomy (RP), brachytherapy (BT), or curative radiotherapy (RT) at Erasmus MC (2009–2023), the Netherlands. Outcomes included TFS, treatment sequences, and medication patterns across treatment lines. **Results**: Median age at diagnosis was 65 years (IQR 61–69) for RP, 68 (62–73) for BT, and 72 (68–76) for RT. At 10 years, TFS was 89% (95% CI: 84.9–94.1) for BT, 85% (95% CI: 83–87) for RT, and 71% (95% CI: 65.7–75.8) for RP. Most patients remained treatment-free, but up to five treatment lines occurred, mainly in patients with low comorbidity scores. Medication profiles reflected treatment-related morbidity: alpha-blocker use increased after BT and RT, while bladder relaxants were common after RP. Comorbidity-related medication use remained low among patients undergoing multiple sequenced treatments. **Conclusions**: These findings highlight the real-world application of multiple secondary treatments after different primary curative therapy options for PCa and associated comorbidity and medication use patterns. They confirm the durability and long-term effectiveness of curative treatments in real-world PCa care. By combining treatment trajectories and medication profiles, RWD provides insights for personalized counseling, helping clinicians and patients anticipate long-term treatment needs, and enabling informed decisions aligned with health status and preferences.

## 1. Introduction

Patients eligible for curative prostate cancer (PCa) treatment can be treated with radical prostatectomy (RP), brachytherapy (BT) or radiotherapy with curative intent (cRT) [1]. These treatments have been well studied in terms of outcomes such as treatment failure, disease progression, quality of life, and prostate cancer-specific mortality (PCSM), often based on (randomized controlled) trials (RCTs) [2,3,4,5]. Although prospective trials are considered the gold standard due to their robust design and methodology, they often include highly selective patient populations that may not fully reflect the broader, more diverse populations encountered in daily clinical practice [6]. Moreover, most trials have limited follow-up (months to years), which does not capture the lifelong nature of PCa, the long-term impact of treatment decisions on survival, and the need for secondary treatments [7]. This limits the generalizability of their findings, as outcomes reported in RCTs may not adequately capture the complexities of real-world treatment and the long-term management of PCa [5]. Additionally, treatment decisions in real-world settings are not always strictly based on clinical guidelines but are influenced by a variety of patient-specific factors, such as comorbidities, age, and personal preferences [8].

The growing availability of electronic health record (EHR) data offers a unique opportunity to address this gap and collect real-life information on outcome data after curative therapy for PCa [9]. Unlike prospective trials, EHR data enables long-term observation across diverse populations. By analyzing patient data from healthcare systems, we can gain insights into real-world treatment trajectories, including treatment-free survival (TFS), treatment sequences, and medication patterns across treatment stages. EHR data, with its high-dimensional nature, allows for a more representative understanding of patient experiences and outcomes across diverse patient groups and as such can be the basis for a more personalized treatment approach.

The aim of this study is to investigate real-world treatment-free survival following initial curative PCa treatment and to analyze the subsequent course of follow-up treatments for PCa patients using data from the Erasmus Medical Center, the largest academic hospital in the Netherlands. This study will contribute to answering the broader questions raised by PIONEER (Prostate Cancer DIagnOsis and TreatmeNt Enhancement through the Power of Big Data in EuRope) regarding long-term care and treatment outcomes in prostate cancer patients [10]. Additionally, this study will explore how real-world data and big data analytics can support clinical decision-making and patient expectations and help shape future individualized prostate cancer treatment strategies.

## 2. Materials and Methods

### 2.1. Study Design and Data Source

This observational study was conducted using electronic health record (EHR) data from the Erasmus Medical Center, the largest academic tertiary care center in the Netherlands. The study included all PCa patients diagnosed between 2009 and 2023, focusing on those who received curative treatment as their first-line treatment. Erasmus MC is the regional referral center for radiotherapy. Prostatectomy was performed in peripheral centers from September 2018.

### 2.2. Patient Identification and Classification

Patients were identified based on a PCa diagnosis recorded in the EHRs. They were classified according to their initial curative treatment, which included RP, cRT, and BT. cRT was defined as five or more sessions administered within a 90-day period. Treatment identification relied on Diagnosis Treatment Combination (DTC (Dutch: ‘DBC’)) codes, a standardized classification system in the Dutch healthcare system.

### 2.3. Exclusion Criteria

The study excluded records before 2009 due to limitations in DTC coding. Communication with data management experts revealed that while DTC coding was introduced in 2004, the reliability of the linkage between procedures and DTC’s performed between 2004 and 2009 was limited.

### 2.4. Data Collection

Baseline patient characteristics at initial PCa diagnosis included age and prostate-specific antigen (PSA) levels from standardized fields in the EHR, while the International Society of Urological Pathology Grade Group (ISUP GG) and tumor/node/metastasis stage (TNM) were extracted from free text fields for each patient using automated code. Results were manually reviewed to ensure accuracy. These characteristics were stratified by initial treatment to provide a detailed overview of the patient population. Because data extraction was fully automated and the resulting dataset was pseudonymized, individual records could not be re-identified to supplement missing values.

### 2.5. Outcome Measures

#### 2.5.1. Treatment-Free Survival

Treatment-free survival (TFS) was the primary outcome measure. TFS is defined as the time from the date of the first curative treatment to the initiation of any secondary treatment. Secondary treatments included androgen deprivation therapy (ADT), chemotherapy (CTX), lymph node dissection (LND), radiotherapy (RT), and radium therapy (RdT). Conservative treatment and palliative care were not classified as active secondary treatments but were included in TFS analysis because their initiation indicates disease progression. The time to event analysis for TFS was measured using Kaplan–Meier survival curves. Restricted mean survival time (RMST) was calculated to assess the average time patients remained treatment-free before requiring secondary treatment or died with a time horizon of 10 years.

#### 2.5.2. Statistical Analysis

Descriptive statistics were used to summarize patient characteristics and treatment patterns. For continuous variables, medians and interquartile ranges (IQRs) were reported. For categorical variables, frequencies and percentages were calculated.

#### 2.5.3. Medication Categorization and Analysis

We evaluated medication use throughout the study period. Medication use was extracted and categorized for each patient, stratified by use >1 year, use between 364 and 1 days before initial treatment, use after initial treatment, and use between subsequent treatments. All prescribed drugs were grouped into predefined therapeutic categories and labeled with the Anatomical Therapeutic Chemical (ATC) classification system (see Supplementary Table S1 for the complete list) [11].

These categories included both medication for treatment-related symptoms and drugs indicative of underlying non-PCa-related comorbidities. To define which medications were reflective of comorbidities, the ATC classification system was used. The ATC classification is used to divide medication in different groups based on the organ or system on which they act. Specifically, medication categories that acted on blood and blood-forming organs (ATC; B), the cardiovascular system (C), or the respiratory system (R) were considered reflective of comorbidities.

We compared medication use before and after treatment to assess changes in prescription patterns over time. Additionally, we examined differences in medication profiles between patients receiving curative treatments and those undergoing subsequent lines of therapy. Transitions in medication use were analyzed across treatment stages. This approach enabled us to characterize evolving medication profiles along the treatment trajectory. Medication use for patients who switched to palliative care is presented for informational purposes only; since this is not considered therapeutic, it was excluded from the overall profile of patients undergoing further treatments.

### 2.6. Ethical Consideration

The study used anonymized patient data obtained from the Erasmus Medical Center’s EHR system, specifically provided through the Research Suite of the Erasmus Medical Center. As this was a retrospective study using anonymized data, patient consent was waived by the Institutional Review Board (IRB), in accordance with ethical guidelines for retrospective research.

## 3. Results

### 3.1. Patient Demographics and Baseline Characteristics

Between 2009 and 2023, a total of 6007 PCa patients were identified. A total of 3024 patients received curative treatment and were included in the analysis, of whom 2240 received cRT, 420 BT, and 364 RP. Median age at diagnosis was 72 years (IQR 68–76) in the cRT group, 68 years (IQR 62–73) in the BT group, and 65 years (IQR 61–69) in the RP group, with a PSA of 8 (IQR 3–17), 9 (IQR 6–16), and 7 (IQR 5–14) ng/mL, respectively (Table 1). The median year for the start of initial treatment was 2017 (IQR 2014–2019) for cRT, 2015 (IQR 2014–2018) for BT, and 2014 (IQR 2014–2019) for RP. The majority of patients had a known ISUP GG at diagnosis of 2 or 3 (42%) and a tumor stage of T2 (36%) or T3 (42%). 

### 3.2. Treatment Patterns

**Figure 1:**
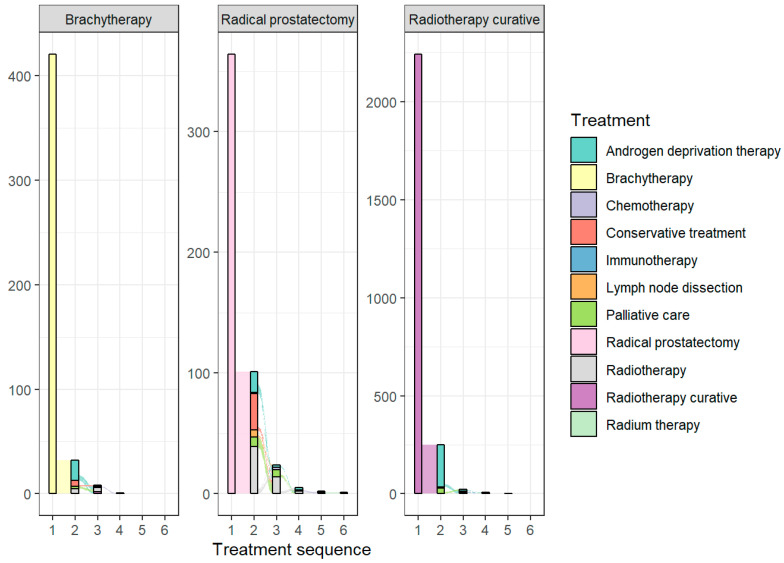
Treatment sequences after initial treatment with curative intent.

Treatment patterns after initial treatment are presented in Figure 1. Results are presented separately for each initial treatment modality.

**Figure 2:**
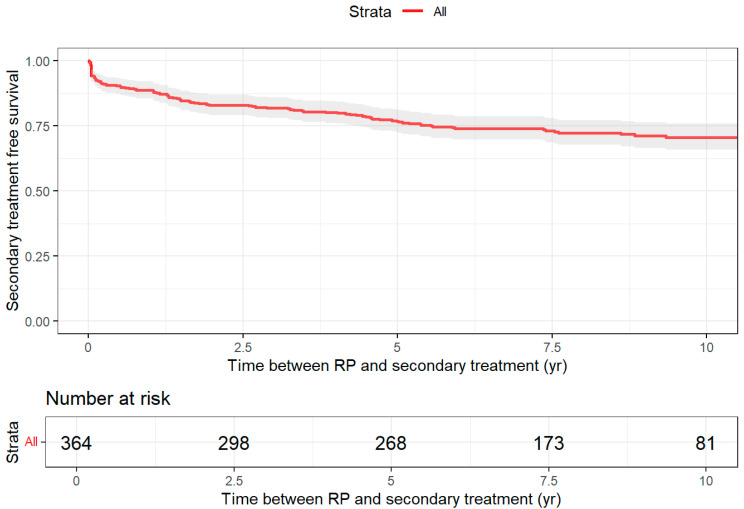
Treatment-free survival after initial radical prostatectomy.

For patients treated with RP, the probability of remaining treatment-free at 10-year FU was 71% (95% CI: 65.7–75.8) (Figure 2). The median follow-up time for patients treated with RP who did not require second-line treatment was 8.7 years (IQR: 6.8–10.5 years). The RMST with a horizon of 10 years was 7.8 years (95% CI: 7.8–8.2). In the second-line treatment setting, RT (n = 39) and conservative treatment (n = 30) were the most commonly used therapies, followed by ADT (n = 17). RT remained dominant in third-line therapy (n = 14). In the fourth-line treatment, ADT and RT were equally represented (n = 2). Up to five additional treatments were recorded after RP.

**Figure 3:**
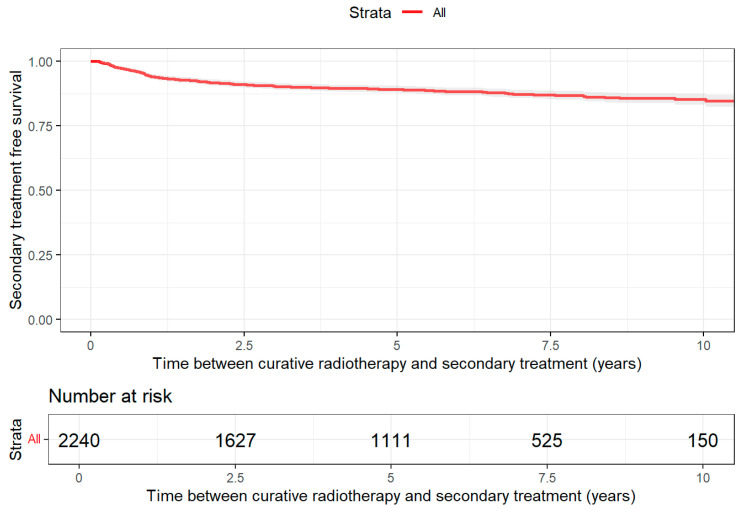
Treatment-free survival after initial radiotherapy with curative intent.

For patients treated with cRT, the probability of remaining treatment-free at the 10-year follow-up (FU) was 85% (95% CI: 83–87) (Figure 3). Patients treated with cRT who did not require second-line treatment had a median follow-up of 5.5 years (IQR: 3.0–7.6 years). The RMST was 9.0 years (95% CI: 8.8–9.1). Among those requiring second-line treatment, ADT was the most frequently used therapy (n = 214), followed by palliative care (n = 29). ADT remained dominant in third-line therapy (n = 7), while CTx was the most common fourth-line treatment (n = 4). A maximum of up to four additional treatments were recorded after cRT.

**Figure 4:**
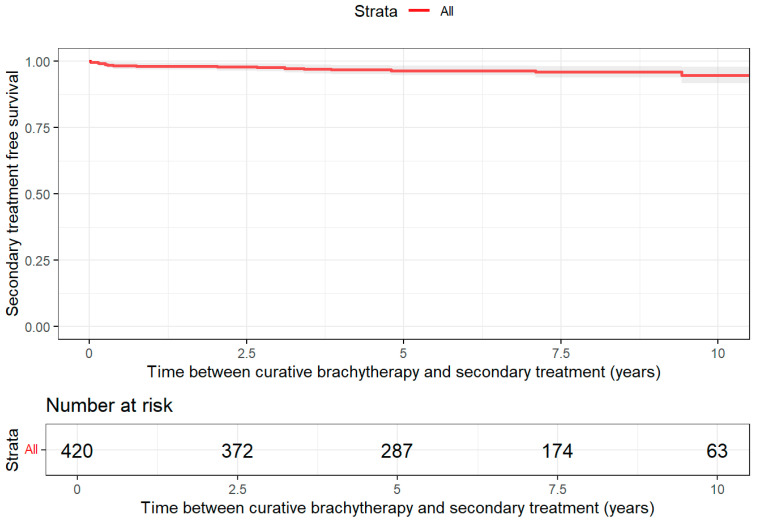
Treatment-free survival after initial brachytherapy.

For patients treated with brachytherapy (BT), the probability of remaining treatment-free at 10-year FU was 89% (95% CI: 85–94) (Figure 4). Nineteen patients required ADT after BT, but further analysis was not possible due to the small sample size. The median follow-up time for patients treated with BT who did not require second-line treatment was 6.5 years (IQR: 4.2–8.6 years). The RMST was 9.4 years (95% CI: 9.1–9.6). In the second-line treatment setting, ADT was the dominant choice (n = 19), followed by conservative treatment (n = 6) and RT (n = 5). RT was the most commonly used treatment in the third-line setting (n = 6). For the fourth-line treatment, only RT was recorded (n = 1). Up to three additional treatments were recorded after BT.

### 3.3. Medication Use and Changes

Comorbidity-specific medication use was very low across all groups, with less than 2% of patients receiving such drugs. Urological medications were rarely used prior to treatment, with alpha-blocker use exceeding 5% only among brachytherapy patients. After initial treatment, alpha-blocker use increased substantially in brachytherapy patients (15%, n = 61) and in those treated with cRT (11%, n = 245). Bladder relaxants were most frequently prescribed to patients undergoing RP (26%, n = 93) and brachytherapy (9.8%, n = 41), but remained low after cRT (2.6%, n = 62).

Among patients initially treated with RP, the largest increases in medication use were observed one month after treatment, particularly for analgesics (59%, n = 213), antibiotics (63%, n = 228), and heparin (57%, n = 206). Other notable increases included antiemetics (30%, n = 110), antihypertensives (11%, n = 40), laxatives (33%, n = 121), proton pump inhibitors (12%, n = 42), cholesterol-lowering agents (7.7%, n = 28), and TAR (7.7%, n = 28). In brachytherapy patients, the most pronounced increases were seen for analgesics (9%, n = 38), antibiotics (11%, n = 47), benzodiazepines (10%, n = 42), and laxatives (8.3%, n = 35), while other medications were prescribed in fewer than 5% of cases. For cRT patients, no medication category exceeded 5% usage.

Comorbidity-associated medication use remained very low among patients who required subsequent lines of therapy. Following a second treatment after radical prostatectomy, only a small proportion of patients received such medication, including antihypertensives (5.4%, n = 5), statins (4.3%, n = 4), antiplatelet therapy (3.2%, n = 3), and vitamin K antagonists (1.1%, n = 1). Among brachytherapy patients, the proportion was slightly higher, with 14% (n = 4) receiving statins and 3.4% (n = 1) receiving antihypertensives or antiplatelet therapy, whereas in the cRT group the prevalence remained below 1% for antihypertensives, antiplatelet therapy, and vitamin K antagonists. With subsequent treatment lines, modest increases were observed. Importantly, only very few patients underwent a third, fourth, or fifth treatment, and therefore the reported percentages should be interpreted with caution. After a third treatment, 14% (n = 3) of patients received antihypertensives, 9.1% (n = 2) statins, and 4.5% (n = 1) diuretics. Following a fourth treatment, 31% (n = 4) were prescribed antihypertensives, while 7.7% (n = 1) received statins and direct oral anticoagulants. After a fifth treatment, 25% (n = 1) of patients were treated with a direct oral anticoagulant. Detailed medication profiles for each treatment modality are presented in Supplementary Table S1A–C.

## 4. Discussion

This study provides real-world insight into long-term treatment patterns following initial curative therapy for PCa in a high-volume tertiary center. The results show that the majority of patients remain treatment-free for extended periods after their primary curative treatment. Specifically, 85% of patients treated with RT, 89% of patients treated with BT, and 71% of patients treated with RP remained free of secondary treatments at 10 years. These findings highlight the long-term effectiveness of initial curative treatments in a real-world, heterogeneous population.

Medication patterns offer insight into treatment-related morbidity. Alpha-blocker use increased substantially after brachytherapy (15%) and cRT (11%), which may suggest higher rates of voiding symptoms in these groups. In contrast, bladder relaxants were most frequently prescribed after RP (26%), consistent with known post-surgical storage symptoms. Analgesics, antibiotics, and heparin were also markedly increased after RP, reflecting the intensity of perioperative care. These findings highlight the importance of considering baseline urinary complaints and comorbidities during counseling, as pre-existing symptoms may worsen depending on treatment modality. Such information can help patients make more informed decisions about their preferred treatment, aligned with their health status and expectations.

While most patients remained treatment-free within our center, a maximum of five lines of therapy were recorded for patients treated with RP, four for patients treated with cRT, and three for patients treated with BT. Among patients undergoing multiple treatment lines, only a small proportion used comorbidity-related medication (9.1–33%), which is far below the expected prevalence in this age group. In the general Dutch population of 75 years and older, 84% have a chronic disease and 59% have more than one chronic condition [12]. This suggests that multi-line treatment pathways are typically initiated only in patients who are relatively healthy. Counseling should therefore set realistic expectations and tailor strategies to patient profiles.

These real-world insights enable clinicians to move beyond guideline averages and provide individualized counseling. For example, older patients with significant comorbidities are unlikely to undergo multiple treatment lines, whereas younger, fitter patients may face a more complex trajectory. Similarly, urinary symptom profiles should inform treatment choice, as RP is associated with higher rates of bladder relaxant use and BT with increased alpha-blocker prescriptions. Integrating these data into shared decision-making can help align treatment strategies with patient preferences and long-term expectations.

Compared to the existing literature, our study offers a broader, real-life perspective. Previous studies, such as that by Yap et al., a population-based study, reported only on initial treatment patterns [13]. Stucki et al. analyzed treatment patterns using US-based healthcare claim data, distinguishing between localized and metastatic PCa and addressing treatment sequences after secondary treatment, although this was limited to patients with metastatic disease [14]. Claims-based approaches differ from the Dutch setting, where no unified insurer-linked database exists, whereas our EHR-based approach provides continuous clinical information across care episodes, enabling reconstruction of complete treatment trajectories. Our findings complement Beyer et al., who examined secondary treatment incidence in localized PCa using PRIAS and ERSPC data within the PIONEER platform [15]. Their study focused on low-risk patients with strict inclusion criteria (T1c–T2, Gleason ≤ 3 + 3, PSA ≤ 10 ng/mL), which likely explains the lower rates of treatment change compared to our heterogeneous, real-world cohort. In contrast, our analysis covers the full spectrum of treatment sequences and associated medication patterns over a 14-year period, providing insights into long-term trajectories and multi-line treatment pathways that were not addressed in previous work. To our knowledge, no prior study has examined these patterns across all curative modalities in a real-world setting [16,17].

Differences in survival outcomes compared to previous reports may reflect cohort selection and recurrence definitions. Peacock et al. reported 10-year event-free survival (EFS) following RP based on the Gleason score, with an overall EFS of 79% and stratified rates of 87% (Gleason 2–6), 74% (Gleason 7), and 52% (Gleason 8–10) [18]. Suárez et al. observed biochemical recurrence rates at 10 years of 24% for RP, 29% for BT, and 43% for EBRT in a cohort with primarily low- to intermediate-risk disease [19]. While direct comparison is challenging due to differences in endpoints and study populations, our observed TFS rates seem to be higher. This may reflect differences in cohort selection, recurrence definitions, or initiation of treatment after recurrence [18,19,20,21]. Our cohort included a real-world, heterogeneous patient population, with median ages at diagnosis of 72, 68, and 65 years for cRT, BT, and RP, respectively, and a majority classified as ISUP grade 2–3 or stage T2–T3. This contrasts with the lower-risk and narrowly selected populations in the abovementioned literature. In addition to population differences, temporal factors within our center may also have influenced outcomes. Specifically, radical prostatectomy was no longer performed at our institution after 2018, whereas radiotherapy and follow-up or subsequent treatments continued throughout the entire study period. These differences in treatment availability across time may introduce era effects, such as evolving diagnostic pathways or changes in surgical practice, which should be considered when interpreting outcome differences.

The use of retrospective RWD and big data approaches in general also has limitations. Treatment initiation is not always directly linked to clinical or biochemical progression; some relapses may remain untreated or undocumented, potentially leading to overestimation of TFS, and patients could have received subsequent treatments at other hospitals, which could lead to incomplete treatment records in our dataset. Additionally, our dataset lacks key clinical variables such as performance status or patient preferences. Finally, a substantial proportion of baseline PSA, ISUP, and TNM values were missing. Because data extraction was fully automated and the analytic dataset was pseudonymized, individual records could not be re-identified or manually supplemented after extraction.

To overcome these limitations, RWD from multiple hospitals would allow for more robust analyses and quality improvement. On a broader scale, collaborative data initiatives such as the European Urology Evidence Hub are promising [22]. By extracting data from multiple countries and healthcare systems, the Evidence Hub enables richer phenotyping across patient populations and treatment contexts. Access to larger, heterogeneous cohorts may enable the development of predictive models tailored to patient-specific goals, such as maximizing TFS, minimizing ADT exposure, or achieving the shortest effective course of care. These insights could enhance shared decision-making by aligning treatment strategies with individual patient values and long-term expectations.

A key strength of our study is the use of structured EHR data collected over a 14-year period from one large academic center in the Netherlands, performing all available curative treatments for PCa. This enabled reconstruction of detailed longitudinal treatment sequences and estimation of real-world TFS across treatment modalities. Compared to prospective cohort studies, our approach is scalable, pragmatic, and more reflective of routine clinical practice. In addition, the use of RWD enables the inclusion of patients with comorbidities, older age, and variable follow-up intensity, thereby enhancing generalizability.

## 5. Conclusions

To our knowledge, this is the first study to examine the full spectrum of prostate cancer treatment following initial curative therapy based on structured EHR data, providing a proof of concept for using real-world data to address knowledge gaps in long-term treatment trajectories. Most patients remained treatment-free for extended periods, while multi-line treatment pathways were observed primarily in relatively healthy patients. These findings enable clinicians to tailor counseling based on patient fitness, urinary symptom profiles, and likelihood of requiring additional treatments. By integrating these insights into shared decision-making, we can offer patients more realistic expectations and support personalized treatment planning.

## Figures and Tables

**Table 1:** Patient characteristics stratified by initial treatment.

Characteristic	Brachytherapy N = 420 ^1^	Radical Prostatectomy N = 364 ^1^	Radiotherapy Curative N = 2240 ^1^
PSA (ng/mL)	9 (6, 16)	7 (5, 14)	8 (3, 17)
Unknown	378	352	2205
Age at diagnosis	68 (62, 73)	65 (61, 69)	72 (68, 76)
Gleason score			
6	134 (56%)	103 (37%)	252 (23%)
7	104 (43%)	133 (47%)	504 (46%)
8	3 (1.2%)	29 (10%)	198 (18%)
9	0 (0%)	15 (5.3%)	133 (12%)
10	0 (0%)	1 (0.4%)	18 (1.6%)
Unknown	179	83	1135
T stage			
T0	4 (1.1%)	2 (0.7%)	2 (0.1%)
T1	125 (33%)	36 (13%)	316 (17%)
T2	194 (52%)	127 (46%)	704 (38%)
T3	50 (13%)	102 (37%)	780 (42%)
T4	2 (0.5%)	10 (3.6%)	56 (3.0%)
Unknown	45	87	382
N stage			
Nx	289 (77%)	191 (69%)	733 (39%)
N0	72 (19%)	48 (17%)	932 (50%)
N+	14 (3.7%)	38 (14%)	193 (10%)
Unknown	45	87	382
M stage			
Mx	288 (77%)	230 (83%)	874 (47%)
M0	85 (23%)	44 (16%)	927 (50%)
M+	2 (0.5%)	3 (1.1%)	57 (3.1%)
Unknown	45	87	382

^1^ n (%); Median (Q1, Q3).

## Data Availability

The data is not publicly available because they consist of anonymized institutional data that cannot be shared due to privacy and institutional restrictions.

## Supplementary Materials

The following supporting information can be downloaded at: https://www.mdpi.com/article/10.3390/jpm16010022/s1, Supplementary Table S1A: Medication use primary treatment, Table S1B: Medication use second line treatment, Table S1C: Medication use third, fourth and fifth line treatment.

## Abbreviations

The following abbreviations are used in this manuscript:

PCa	Prostate cancer
RWD	Real-world data
EHR	Electronic health records
EFS	Event-free Survival
TFS	Treatment-free survival
RP	Radical prostatectomy
BT	Brachytherapy
RT	Radiotherapy
cRT	Curative radiotherapy
ADT	Androgen deprivation therapy
CTX	Chemotherapy
LND	Lymph node dissection
RdT	Radium therapy
RMST	Restricted mean survival time
PSA	Prostate-specific antigen
ISUP GG	International Society of Urological Pathology Grade Group
TNM	Tumor/Node/Metastasis
DTC	Diagnosis Treatment Combination
ATC	Anatomical Therapeutic Chemical classification system
FU	Follow-up
PCSM	Prostate cancer-specific mortality
RCT	Randomized controlled trial
IRB	Institutional Review Board
SIOG	International Society of Geriatric Oncology
PRIAS	Prostate Cancer Research International Active Surveillance
ERSPC	European Randomized Study of Screening for Prostate Cancer
PIONEER	Prostate Cancer DIagnOsis and TreatmeNt Enhancement through the Power of Big Data in EuRope
