# Supplementary material for: Treatment-Free Survival and the Pattern of Follow-Up Treatments After Curative Prostate Cancer Treatment, a Real-World Analysis of Big Data from Electronic Health Records from a Tertiary Center
Source: J Pers Med. 2026 Jan 4;16(1):22. doi: 10.3390/jpm16010022 (PMC12843471; doi:10.3390/jpm16010022)
Supplement: Supplementary file 1 [file jpm-16-00022-s001.zip › jpm-4046778-supplementary.pdf]

Supplementary Table S1A; Medication use primary treatment

**Medication category**	**ATC code**	**BT** (n = 420)		**RP** (n = 364)		**cRT** (n = 2240)	
		Pre-treatment	Post-treatment	Pre-treatment	Post-treatment	Pre-treatment	Post-treatment
5-Alpha Reductase Inhibitor	G			1 (0.3%)		1 (<0.1%)	
Alpha Blocker	G	22 (5.2%)	61 (15%)	3 (0.8%)	12 (3.3%)	51 (2.3%)	245 (11%)
Alpha Blocker / 5-Alpha Reductase Inhibitor	G					5 (0.2%)	7 (0.3%)
Antibiotics	J	17 (4.0%)	47 (11%)	16 (4.4%)	228 (63%)	466 (21%)	15 (0.7%)
Antiemetics	A		9 (2.1%)	7 (1.9%)	110 (30%)	2 (<0.1%)	4 (0.2%)
Antihypertensives	C	1 (0.2%)	5 (1.2%)	7 (1.9%)	40 (11%)	11 (0.5%)	2 (<0.1%)
Antipsychotics	N			1 (0.3%)	3 (0.8%)		
Benzodiazepines	N	4 (1.0%)	42 (10%)	1 (0.3%)	18 (4.9%)	5 (0.2%)	2 (<0.1%)
Antimuscarinics	G	5 (1.2%)	41 (9.8%)		93 (26%)	7 (0.3%)	62 (2.8%)
Lipid-Lowering Agents	C	1 (0.2%)	5 (1.2%)	3 (0.8%)	28 (7.7%)	10 (0.4%)	0 (0%)
Corticosteroids	H		1 (0.2%)	1 (0.3%)	2 (0.5%)	5 (0.2%)	8 (0.4%)
Topical Corticosteroid Cream	A						1 (<0.1%)
Antidiarrheal Agents	A			1 (0.3%)	1 (0.3%)	1 (<0.1%)	29 (1.3?%)
Diuretics	C		1 (0.2%)		3 (0.8%)	1 (<0.1%)	
Direct Oral Anticoagulant	B					1 (<0.1%)	
Intravenous Glucose	V		1 (0.2%)		1 (0.3%)		
Heparin	B	1 (0.2%)	17 (4.0%)	30 (8.2%)	206 (57%)	1 (<0.1%)	2 (<0.1%)
Inhalation Medication	R		1 (0.2%)	2 (0.5%)	8 (2.2%)		
Laxatives	A	8 (1.9%)	35 (8.3%)	1 (0.3%)	121 (33%)	44 (2.0%)	60 (2.7%)
Proton Pump Inhibitor	A	3 (0.7%)	2 (0.5%)	3 (0.8%)	42 (12%)	10 (0.4%)	8 (0.4%)
Analgesics	N	4 (1.0%)	38 (9.0%)	10 (2.7%)	213 (59%)	6 (0.3%)	16 (0.7%)
Thrombocyte Aggregation Inhibitor	B	2 (0.5%)	2 (0.5%)	7 (1.9%)	28 (7.7%)	8 (0.4%)	1 (<0.1%)
Vitamin K Antagonist	B			1 (0.3%)	5 (1.4%)	1 (<0.1%)	

ATC = Anatomical Therapeutic Chemical code; A = Alimentary tract and metabolism; B = Blood and blood forming organs; C = Cardiovascular system; G = Genito-urinary system and sex hormones; H = Systemic hormonal preparations (excl. sex hormones and insulins); J = Anti-infectives for systemic use; N = Nervous system; R = Respiratory system; V = Various; BT = Brachytherapy; RP = Radical Prostatectomy; cRT = Curative Radiotherapy; Pre-treatment = 365 till 1 day before treatment; Post-treatment = one month after treatment.

Supplementary Table S1B; Medication use second line treatment

**Medication category**	**ATC code**	**Second line treatment**												
		**BT**			**RP**						**cRT**			
		**ADT** (n=19)	**Cons** (n=6)	**RT** (n=5)	**ADT** (n=17)	**ChX** (n=1)	**Cons** (n=30)	**LND** (n=6)	**Pal** (n=8)	**RT** (n=39)	**ADT** (n=214)	**ChX** (n=1)	**LND** (n=2)	**Pal** (n=29)
5-Alpha Reductase Inhibitor	G	6 (31%)		1 (20%)	1 (5,8%)						46 (21%)		1 (50%)	1 (3,5%)
Alpha Blocker / 5-Alpha Reductase Inhibitor	G	1 (5,3%)									6 (2,8%)			
Antibiotics	J	9 (47%)	1 (17%)	3 (60%)	9 (53%)	1 (100%)	3 (1,0%)	4 (67%)	5 (63%)	26 (66%)	1 (0,05%)			
Antiemetics	A	3 (16%)			3 (18%)	1 (100%)	3 (1,0%)	3 (50%)	1 (13%)	12 (31%)	4 (1,9%)			
Antihypertensives	C			1 (20%)					1 (13%)	4 (10%)	1 (0,05%)	1 (100%)		
Antipsychotics	N			1 (20%)	1 (5,8%)					1 (2.6%)	1 (0,05%)			
Benzodiazepines	N	5 (26%)	2 (33%)	3 (60%)	1 (5,8%)				1 (13%)	1 (2.6%)				
Antimuscarinics	G	7 (37%)	1 (17%)	3 (60%)	2 (12%)		5 (45%)		3 (38%)	18 (46%)	16 (7,4%)			
Lipid-Lowering Agents	C	3 (16%)	1 (17%)		1 (5,8%)			1 (17%)	1 (13%)	1 (2.6%)			1 (50%)	
Corticosteroid	H										4 (1,9%)			
Antidiarrheal Agents	A	1 (5,3%)									8 (3,7%)			
Heparin	B	6 (31%)	1 (17%)	3 (60%)	6 (35%)		1 (9.1%)	2 (33%)	3 (38%)	12 (31%)	1 (0,05%)			
Laxatives	A	5 (26%)		3 (60%)	5 (29%)		2 (18%)	1 (17%)	2 (25%)	17 (44%)	13 (6,1%)	1 (100%)		3 (10%)
Proton Pump Inhibitor	A	2 (11%)	1 (17%)	2 (40%)	2 (12%)			1 (17%)	1 (13%)	4 (10%)	2 (0,09%)	1 (100%)		
Analgesics	N	6 (31%)	3 (50%)	3 (60%)	8 (47%)	1 (100%)	6 (55%)	3 (50%)	6 (75%)	23 (59%)	6 (2,8%)	1 (100%)		1 (3,5%)
Thrombocyte Aggregation Inhibitor	B	1 (5,3%)						1 (17%)		2 (5.1%)	1 (0,05%)		1 (50%)	
Vitamin K Antagonist	B									1 (2.6%)	1 (0,05%)			
Chemotherapy	L										1 (0,05%)			
Detoxification	L										1 (0,05%)			

ATC = Anatomical Therapeutic Chemical code; A = Alimentary tract and metabolism; B = Blood and blood forming organs; C = Cardiovascular system; G = Genito-urinary system and sex hormones; H = Systemic hormonal preparations (excl. sex hormones and insulins); J = Anti-infectives for systemic use; L = Antineoplastic and immunomodulating agents; N = Nervous system; BT = Brachytherapy; RP = Radical Prostatectomy; cRT = Curative Radiotherapy; ADT = Androgen Deprivation Therapy; RT = Radiotherapy; ChX = Chemotherapy; LND = Lymph Node Dissection; Cons = Conservative treatment; Pal = Palliative treatment.

Supplementary Table S1C; Medication use third, fourth and fifth line treatment

Medication category	ATC code	Thirth line treatment						Fourth line treatment					Fifth line treatment		
		ChX (n=9)	IT (n=3)	RT (n=46)	RaT (n=3)	ADT (n=10)	Pal (n=10)	ChX (n=5)	RT (n=4)	IT (n=1)	RaT (n=3)	ADT (n=2)	RaT (n=1)	ChX (n=1)	Pal (n=1)
Alpha Blocker	G	1 (11%)	1 (33%)			2 (20%)		1 (20%)			2 (67%)				
Antibiotics	J	1 (11%)	1 (33%)	2 (4,3%)				2 (40%)		1 (100%)	2 (67%)	1 (50%)			
Antiemetics	A	1 (11%)	3 (100%)	2 (4,3%)	1 (33%)	1 (10%)	1 (10%)	2 (40%)	1 (25%)	1 (100%)					
Antihypertensives	C	1 (11%)			2 (67%)	1 (10%)					1 (33%)				
Antipsychotics	N									1 (100%)					
Antimuscarinics	G		1 (33%)				1 (10%)								
Lipid-Lowering Agents	C	1 (11%)				1 (10%)		3 (60%)		1 (100%)	2	1 (50%)	1 (100%)	1 (100%)	
Corticosteroids	H	3 (33%)	2 (67%)	1 (2,2%)	1 (33%)	1 (10%)	1 (10%)				1 (33%)				1 (100%)
Antidiarrheal Agents	A	1 (11%)									1 (33%)				1 (100%)
Direct Oral Anticoagulant								2 (40%)	1 (25%)	1 (100%)	2 (67%)	1 (50%)			
Diuretics	C	1 (11%)													
Heparin	B	1 (11%)		2 (4,3%)											
Laxatives	A	2 (22%)	1 (33%)	2 (4,3%)	1 (33%)	1 (10%)	1 (10%)	1 (20%)	1 (25%)	1 (100%)	1 (33%)			1 (100%)	
Proton Pump Inhibitor	A	4 (44%)		1 (2,2%)	1 (33%)	2 (20%)		2 (40%)	1 (25%)	1 (100%)	2 (67%)				
Analgesics	N	1 (11%)	1 (33%)		2 (67%)	1 (10%)	1 (10%)								
Thrombocyte Aggregation Inhibitor	B			1 (2,2%)											

ATC = Anatomical Therapeutic Chemical code; A = Alimentary tract and metabolism; B = Blood and blood forming organs; C = Cardiovascular system; G = Genito-urinary system and sex hormones; H = Systemic hormonal preparations (excl. sex hormones and insulins); J = Anti-infectives for systemic use; N = Nervous system; ADT = Androgen Deprivation Therapy; RT = Radiotherapy; ChX = Chemotherapy; Pal = Palliative treatment; IT = Immunotherapy; RaT = Radium Therapy
